# Salivary nitric oxide level and total antioxidant activity in saliva samples collected by different techniques

**DOI:** 10.1186/1753-6561-8-S4-P83

**Published:** 2014-10-01

**Authors:** Allisson Justino, Adriele Souza, Olga Bocanegra, Renata Teixeira, Foued Espindola

**Affiliations:** 1 Laboratory of Biochemistry and Molecular Biology. Institute of Genetics and Biochemistry, Federal University of Uberlândia, Uberlândia, Brazil

## Background

Ideal biomarkers have the ability to identify a physiological disorder with a high degree of accuracy [1]. The research involving saliva enable the discovery of biomarkers such as proteins and a variety of molecules that act as indicators of pathophysiologic state [2]. Nitric Oxide is a biomarker related with oxidative stress and it is an important hemodynamic regulator in physical activity [3]. The antioxidant activity is responsible for the body's defense mechanisms against damage caused by free radicals and its measurement is important to provide information about the biological system [4]. Saliva collection devices are commercially available, featuring their easy handling, and are adequate for the rapid and standardized collection of unstimulated and stimulated saliva. This study analyzed the profile of salivary nitric oxide and total antioxidant activity in relation to different collection methods.

## Methods

This study was approved by the Ethics Committee in Human Research (CEP) of the Federal University of Uberlândia. Saliva samples from 10 healthy subjects were collected by unstimulated methods, with and without saliva accumulation in the mouth, and stimulated methods using Salivette^®^, Parafilm^® ^and chewing gum with mint flavor. Salivary nitric oxide was quantified by a colorimetric method using Griess reagent. The colorimetric method FRAP (Ferric Reducing Antioxidant Power) was used to measure the total antioxidant activity of the samples. The paired t-test statistical analysis was used to verify the significant difference between the samples analyzed (p < 0.05).

## Results and conclusions

Nitric oxide concentration did not show significant difference (p > 0.05) independent of the method used for stimulated saliva collection. Nitric oxide concentration was significantly higher in unstimulated method with saliva accumulation in the oral cavity (127,06 ± 70,76 µM/mL.min^-1^) compared to unstimulated collection without fluid accumulation (74,42 ± 69,38 µM/mL.min^-1^), and to the three processes with salivary stimulation (34,59 ± 24,85, 40,71 ± 24,30 and 21,43 ± 13,31 µM/mL.min^-1^, using Salivette^®^, Parafilm^® ^and chewing gum, respectively). Furthermore, total antioxidant activity was significantly higher in the unstimulated methods (average 245,87 µM trolox eq/mL.min^-1^) in relation to the stimulated techniques (average 89,28 µM trolox eq/mL.min^-1^). Samples collected using chewing gum with mint flavor showed the lowest total antioxidant activity (39,41 ± 15,36 µM trolox eq/mL.min^-1^), followed by Salivette^® ^collection (98,63 ± 68,97 µM trolox eq/mL.min^-1^). This may indicate that the stimulation process affect the secretion of antioxidant molecules in saliva or their antioxidant activity in the analyzed fluid. The observed differences between the techniques can be explained by the fact that collection mechanisms have influence in the origin and production route of this biological fluid and, consequently, in the concentration and activity of these biomolecules. It is important to clarify that the total volume of saliva collected was considered in the analyses to standardize the saliva dilution in all methods used. Thus, the quantification of some salivary biomarkers such as nitric oxide and total antioxidant activity may contribute to the elucidation of their profile in a particular collection method, helping to choose the technique that best suits for the researcher.
